# A teaching design of ecological class based on immersive virtual reality spatial fusion

**DOI:** 10.3389/fpsyg.2022.874101

**Published:** 2022-07-22

**Authors:** Wenyuan Liu

**Affiliations:** Foreign Languages Institute, Tianjin Normal University, Tianjin, China

**Keywords:** teaching design, IVR, IVRTM, spatial fusion, ecological class

## Abstract

This study investigated the effectiveness of the teaching design scheme called the Immersive Virtual Reality Teaching Model (IVRTM) on a small learner population at the researcher’s university. The study was based on one-term experimental teaching among the undergraduates. An ecological class was set up, extending classroom teaching to 3-layer teaching space, including a physical layer (classroom-based), an information layer (internet-based), and a virtual-reality layer (VR-based). The research aimed (1) to create an online-offline spatial ecological learning environment for higher learning efficiency with a series of learning activities, (2) to uncover the learning effectiveness of VR assistance in oral English, and (3) to acquire the students’ attitudes toward the IVRTM. The study approaches involved literature review, empirical method, questionnaire, interviews, and data analysis. The findings would have a positive significance for the promotion of new technology applications in SLA teaching and provide novel and preliminary references for teachers pursuing an effective teaching design of ecological classes based on IVR spatial fusion.

## Introduction

Digital technology is helpful to stimulate learners’ visual, auditory, and tactile perceptions for improving their learning efficiency (Yang and Gou, 2020). It could promote students’ comprehensive literacy in education, such as autonomous learning ability, cognitive ability, and thinking ability (Zhang, 2011). Big Data has been used widely in some aspects of education, such as academic performance evaluation, education policy guidance, and personalized demand according to data analysis (Jayashankar, 2018). Data analysis enables teachers to comprehensively evaluate students’ academic performance, giving them feedback and instructing them in personalized learning (Zhang and Li, 2013). Online–offline fusion teaching is also booming in China, especially in the post-epidemic era of COVID-19. Currently, immersive virtual reality convergence technology is increasingly developing as a tool for education and promoting educational innovation (Hu et al., 2021). Zhen (2017) attempted to construct a model of online–offline ecological English classes in high schools in China. Many researches have been conducted from the perspective of VR technology itself. Currently, some educators begin to have some VR teaching experiments, which are still in their infant stage and there are few research results in this field, not to mention the combination of online–offline fusion with VR assistance. The study made significant contributions to the attempt of three-dimensional multi-spatial fusion teaching combining class-based, internet-based, and VR-based spaces through its unique teaching design, which has a novel and positive significance for the promotion of new technologies in education.

The Immersive Virtual Reality Teaching Model (IVRTM for short) conducted in this research aimed to create an online–offline ecological class with VR assistance for students. An ecological and spatial fusion class was set up in the IVRTM, extending classroom teaching to three-layer multi-space, including classroom teaching, online teaching, and VR- assisted teaching. VR-assisted teaching as part of the IVRTM was adopted in oral English practice. The exploratory study aimed (1) to create an online–offline multi-spatial ecological learning environment for higher learning efficiency with a series of learning activities, (2) to uncover the learning effectiveness of VR assistance in oral English, and (3) to acquire the students’ attitudes toward the IVRTM. The theoretical and empirical methods combined together compose the research framework of this paper, including literature review, empirical method, questionnaire, interviews, and data analysis. The IVRTM as a teaching model in SLA is based on Multimodal Learning Theory (Wang and Mao, 2021) and Production-Oriented Approach (POA for short) (Wen, 2015). To test the effectiveness of the IVRTM, an empirical method was conducted and 109 students were recruited randomly to participate in the teaching experiment over a term. Class 1 as the control group was instructed by the traditional teaching model without a digital learning platform and VR technology support, and Class 2 as the experiment group followed the IVRTM with the VR and learning platform assistance both digitally and technologically. The students in two were asked to complete a variety of teaching activities according to the teaching plan. The final exam results were analyzed to test the significance of the IVRTM. Apart from it, data analyses of the questionnaire and Big Data on the learning platform were adopted to prove the hypotheses of the research. The findings, limitations, and suggestions were discussed at the end of the thesis. The findings would have a novel and positive significance for the promotion of new technological applications in SLA teaching and provide preliminary references for teachers pursuing an effective teaching design of ecological class based on IVR spatial fusion.

## Literature review

According to the existing research and the research protocol, this study employed a systematic and comprehensive review approach to search, select, and analyze the relevant literature published in recent years to achieve the research goal. The research is based on the following two theories, one is Multimodal Learning Theory (Wang and Mao, 2021), and the other is Production-Oriented Approach (POA for short) put forward by Wen (2015). VR-assisted teaching is applied to the pilot study.

### Multimodal theory

The rapid development of artificial intelligence and wearable devices not only promotes educational innovation but also contributes to the application of multimodal theory in teaching research. Typically, humans perceive information and pass it to the brain through multiple senses. Each perceptual source or media form is called “mode.” Modality is the communication between human beings through various senses and the external environment. One sensory communication is called a single modality. And three or more are called multimodality, which refers to the resources of discourse symbols that encode meanings with multiple symbolic modes simultaneously (Wang and Mao, 2021). Modes are experienced in different ways by each of the senses – usually visual, auditory, and tactile, which often interact with each other, creating a dynamic learning experience for students. For instance, an educational video might include speech, image, music, and text – all of which can enhance a student’s learning experience. The multimodal theory is to study multimodal learning that makes full use of various human perceptions to optimize the learning experience in a fusion environment (Wang and Mao, 2021). It can also refer to texts that conform to the discourse specification with images, charts, etc., to perform multiple encoding to achieve meaning and people could transfer through various ways including voice, text, gesture, and other modes of communication between human beings through various senses and external environment. Multimodality emphasizes the use of pictures, videos, gestures, body language, and other different modes to convey different knowledge and stimulate learners’ multisensory channels so as to meet the needs of students in language, and knowledge, and thinking (Milicevic et al., 2017). In language teaching, it is usually presented in a synergy of interaction, innovation, and dynamic process involved in multimodal senses of auditory, visual, and tactile modes, including image, gesture, speech, writing, models, spatial, and bodily codes (Zhang and Li, 2013). Some methods commonly used in multimodal theory include phonetics of speech, video clips, physiological tests, eye-tracking records, and digitized logs for deeper insight into learners’ behaviors, cognition, and motivation. The researcher obtains broad evidence according to the learners’ learning behaviors, styles, emotions, and modes. And multimodal data can be collected and analyzed for understanding learners’ learning process. Educators combine the empirical paradigm study with multimodal analytics to obtain continuous, embedded, and real-time feedback on ongoing learning through data (Zhang and Li, 2013; Milicevic et al., 2017). The findings of data analysis are used for reasoning and decision-making in educational reform (Wang and Mao, 2021).

The IVRTM was put forward in this research based on the multimodal theory. Multimodal modes are triggered through a series of online- offline teaching activities as a result of learning efficiency improvement (Zhan, 2019). At present, studies lack multimodal teaching research on spatial fusion classes with VR assistance. The IVRTM teaching aims for mobilizing students’ multisensory perceptions for better learning effectiveness with VR assistance. The study aims to stimulate and activate learners’ multisensory channels in a highly immersive virtual reality setting (Anua et al., 2022). Students employ their information-processing sensory modes in the IVR learning environment. In the teaching scenario, a quantity of multimodal learning data from VR-assisted training can be captured, fused, and analyzed, which verified the learning effect through a comparison with multimodal data between the control class and experiment class and better understood the learning process so as to take some positive measures to intervene teaching if necessary (Hu et al., 2021). According to the research, educators would have more reliable references for processual evaluation and comprehensive evaluation of students’ performance and students could get continuous, embedded and real-time feedback on their ongoing learning (Zhang, 2011). VR-assisted teaching research is a support for the multimodal theory, which will be potential for understanding procedural learning in the future.

### Production-oriented approach *theory*

**FIGURE 1:**
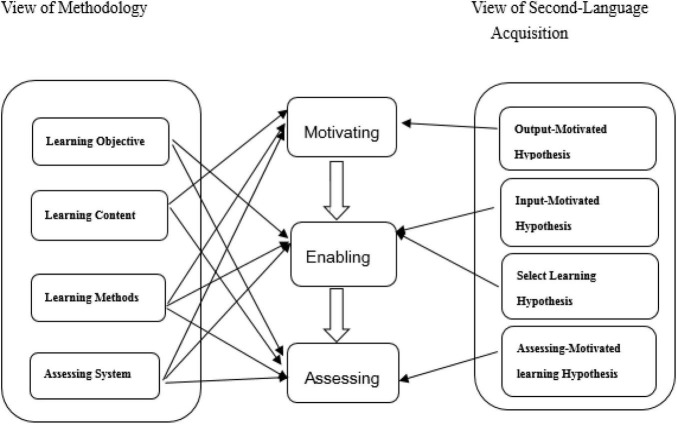
The POA theoretical system.

The Chinese scholar (Wen, 2015, 2018) created the theory of Production–Oriented Approach (POA) based on her early theoretical research on Output-Driven Hypothesis (Wen, 2015, 2018) and Output- Driven Input Facilitation Hypothesis (Wen, 2015, 2016). Wen (2015, 2016, 2021) also set up the POA theoretical system from the perspectives of methodology and SLA. The POA theoretical system had three parts including teaching design, teaching procedures, and theoretical hypotheses (Wen, 2015, 2016, 2018; Figure 1).

The first part was the guiding ideology of teaching in the view of methodology, which consisted of learning objective, learning content, learning method, and assessing system. The second part is composed of three basic steps to achieve the POA teaching objectives motivating, enabling, and assessing. Basically, there were a number of “motivating- enabling-assessing” cycles in the teaching procedures (Wen, 2015, 2016, 2018). Wen (2018) elaborated on the POA tasks and requirements of teaching procedures and proposed evaluation standards for each procedure. Four hypotheses were put forward as theoretical bases and concepts from the perspective of SLA. They were Output-Motivated Hypothesis, Input-Motivated Hypothesis, Select Learning Hypothesis, and Assessing-Motivated Learning Hypothesis (Cumming, 2001; Marsh and Boag, 2013; Wen, 2015, 2021). SLA depended on learning objectives, learning contents, learning methods, and assessing systems motivated by a series of teaching activities and input and output tasks (Rubin, 2005; Zhu and Bai, 2019). Motivating, enabling, and assessing are closely relevant, but independent of each other, acting together to contribute to students’ comprehensive language competence (Wen, 2015, 2016, 2018).

**FIGURE 2:**
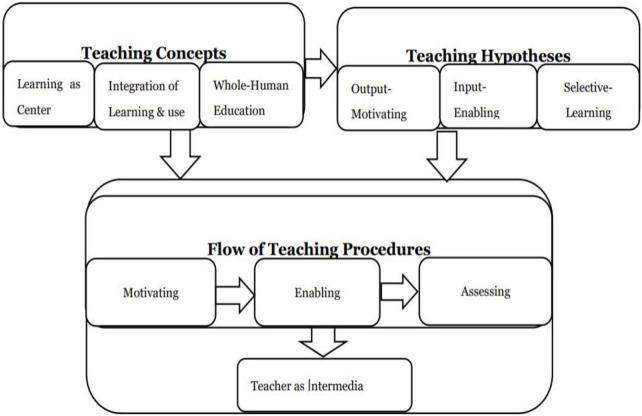
Relations of three parts in POA system.

On the other hand, the POA theory involves three educational philosophies including Learning as Center, Integration of Learning and Use, and Whole-Human Education. Learning as Center indicates that learning is the basic and core behavior in the learning and teaching process. By contrast, students’ core status in the teacher-centered class may be neglected, whereas teachers’ leading role in student-centered class tends to be ignored in practice (Wen, 2015, 2016). The concept of Learning as Center emphasizes that students and teachers should corporate equally and complete a variety of activities and tasks cooperatively in order to achieve teaching objectives. That is to say, both teachers and students are equally important in teaching, and they play equal roles in the teaching and learning process. Integration of Learning and Use advocates for students to improve their language competence through language input and output (Zhan, 2019; Zhu and Bai, 2019). According to the students’ academic proficiency and learning styles, teachers assign them some personalized and differentiated input and output tasks for practice (Fiore and Rosenquest, 2010; Hu et al., 2021). Learning style as one of the determinants is non-linear, dynamic, interactive, and open, enabling students to complete personalized tasks independently. The goal of Whole-Human Education emphasizes the whole growth of students instead of focusing on their specific experience or mastering some skills themselves only. Therefore, the POA theory emphasizes cultivating students’ comprehensive literacy and balanced development regarding cognition, emotion, and quality. The following Figure 2 shows the relations of three parts among the teaching concepts, the teaching hypotheses, and the teaching procedures (Wen, 2015).

The POA theory has been accepted and welcomed by a great many university teachers in China. These teachers could understand and accept the learning-centered concept of the theory and had some fruitful teaching research, such as some teaching designs and empirical experiments under the guidance of the POA theory (Li and Fan, 2018). And a series of motivating-enabling-assessing teaching cycles were created and carried out in the teaching designs. Some preliminary findings have initially shown positive results from the POA teaching experiments (Yang and Gou, 2020). Wang et al. (2021) made some research on motivating, enabling, and assessing, respectively, in teaching and proved that the motivating-enabling- assessing procedures were connected to each other without any strict bounds in the teaching process. They fully affirmed its theoretical advantages in teaching design and adaptation of textbook, especially in the promotion of learning motivation and goal achievements (Zhan, 2019). Moreover, the POA theory has an influence on the international academic circle as well. Wen (2016) made a report on the application of the POA theory for Chinese adults’ SLA in China’s English teaching at the 7th International Conference. And she completed the 7th chapter – *The Production-Oriented Approach*: *A Pedagogical Innovation in University English Teaching in China* in the book of Students, Teachers and Pedagogy edited by Wong and Hyland in 2017. In May of the same year, the POA team held a high-end forum to introduce the theory among the top overseas scholars in Beijing. In October, they also held some international academic seminars to discuss the feasibility of the POA theory throughout the world. And extensive attention was paid to it by the experts attending the seminars. Generally, the POA theory has been already used by more and more Chinese college teachers and is gradually known and accepted by more language experts, scholars, and educators throughout the world.

### Virtual-reality application in the immersive virtual reality teaching model

As an immersive learning experience, VR-assisted teaching makes it possible with the help of digital animation, interactive images, and wearable devices. Basically, there are two main categories of IVR devices. One is a large fixed visual device with a circular wall display with high cost often used in large projects. The other is a kind of convenient, portable, wearable, and hand-held equipment with visual recognition function, by which learners can interact with simulation characters in the immersive virtual space. Generally, the latter is recommended by creating a smart class.

Virtual-reality used in SLA teaching could activate students’ multisensory modes, especially in the immersive human-computer interaction through listening, speaking, reading, and writing in oral English practice. The research on VR technology has been achieved greatly, and more attention to VR teaching is paid by more and more educators (Duan, 2019). The research of VR-assisted online–offline fusion English teaching is still few. The IVRTM in this research creates a three-layer learning environment for students between virtuality and reality in an attempt to higher learning effectiveness by stimulating students’ multisensory modes, such as visual, auditory, and tactile perceptions. The VR-assisted teaching focuses on the students’ experiential learning in the learning process. Smart Room has two sections for computer desktop training and field training, which are open regularly. Before class, the teacher assigns communicative tasks according to the teaching objective. Students first had desktop training to learn and imitate some communicative situations through IVR videos and then experienced them in the training field before class. In class, the teacher would divide the students into several groups and ask them to practice the related communicative tasks in reality. After class, students could practice more in the training field of Smart Room if they would like to. Take the topic “Asking the Way” for example, the students could choose different destinations of communicative tasks, learn the relevant words and sentence patterns first, and then invite a virtual figure online to make a catechetic dialogue. Their speeches could be recorded and kept for later oral improvement and evaluation of pronunciation, intonation, fluency, and accuracy. English teaching aims for not only mastery of the language, but also its humanistic qualities, such as culture, cultural awareness, cultural adaptation, and humanistic spirits. The purpose of oral practice among peers in the traditional teaching aimed for sentence pattern usage, whereas VR-assisted oral practice could show students appropriate speech, culture diffusion, and different ways of thinking. Specifically, language course has the instrumental and humanistic goals to cultivate students’ comprehensive literacy based on the goal of whole-human education. All teaching activities are learning-centered and closely linked input to output. Students searched for input-driven materials by output objective and did the task-driven exercises to improve learning efficiency. In fact, there is no boundary between input and output, and “learning” and “use” are integrated into teaching and learning, which followed the POA philosophy of Integration of Learning and Use (Zhu and Bai, 2019; Chacon et al., 2021). Multiple evaluation methods would be also employed in the oral test, such as machine evaluation, teacher’s evaluation, peer’s mutual evaluation, and self-evaluation. Students as peer assessors could also learn from each other’s advantages and disadvantages. The joint assessment among machine, student, and teacher is worth recommending especially in the spoken and writing evaluation.

Currently, online-offline blended teaching has been promoted extensively in China in the past several years because of COVID-19. More and more universities began to pay attention to the application of VR technology in English teaching, but multimodal and multi-spatial fusion teaching in SLA was still lack of research and practice (Yang and Gou, 2020). And there is even less research on VR-assisted online-offline fusion teaching. Students have a totally different learning experience in a VR-assisted ecological fusion environment. Virtual Lab/Smart Room is one of the most important IVR experiment classes and its display and interaction with real-time feedback can increase students’ learning interest and enthusiasm (Xie, 2019). More virtual teaching experiments in the IVR lab will be conducted and some related curriculum projects will be developed and have broad prospects in the future (Hu et al., 2021).

## Research methodology

The related literature of the IVRTM was reviewed in the previous parts concerning the theoretical framework of the research. The research methodology was discussed further in detail in this part, including research questions, participants, research methods, instruments, and data statistics analysis to clarify the fact that the IVRTM with new technologies is better than the traditional teaching method for higher learning efficiency, and the acceptability of the novel teaching model in the ecological environment based on the spatial fusion would be proved—which are elaborated upon below.

### Research questions

According to the previous literature review and some related theories, it was a fact that few studies have been conducted on the combination of online-offline fusion teaching with VR assistance. Therefore, the IVRTM was put forward by the researcher, aiming for a more effective English teaching model with new technologies. Three research questions would be explored in this study as follows:

(1) Is there any effect of the IVRTM or it is better than the traditional teaching method?
(2) Can students’ oral English practice with VR assistance promote students’ communicative ability?
(3) What are the students’ attitudes toward the IVRTM?

### Participants

The number of 109 participants was recruited randomly from first-year undergraduates from the university at which the researcher worked. Class 1 (*n* = 53) as the control group was instructed by the traditional teaching method without internet-supported learning platforms and VR assistance, but they shared the same teaching plan with the same periods, pre-class warm-up questions, discussion topics, and role-plays, and after-class assignments. Class 2 (*n* = 56) followed a different teaching method—IVRTM, an interactive hybrid teaching method with the support of internet-supported learning platforms and VR technology.

### Methods

This study adopted the research method of teaching empirical study between Class 1 (control group) and Class 2 (experiment group) for a term. They were distributed to the basic-level English classes according to their English scores on the College Entrance Examination (CEE) under 90 marks (150 marks for perfect scores). Their CEE scores were collected and analyzed as a pre-test of their English mastery level before the teaching experiment. After one-term’s experiment, the scores of their final exams were recorded and analyzed as post-test to examine the significance of the experiment results. The same teacher taught the two classes bilingually, half Chinese and half English. The research lasted one term, including 13 weeks, 12 weeks for teaching, and 1 week for the final exam. The teaching plan includes 4 periods (45 min/period) for in-class presentation and 2 periods for pre-class and after-class autonomous learning in each module, completion of six modules per term, and 120 min for the final exam (Table 1).

**TABLE 1:** Comparison of teaching plan in two classes.

	Periods	Teaching plan	Text-book	Final Exam	Teacher	VR assistance	Superstar platform	Teaching methods
Class 1	Same	Same	Same	Same	Same	No	No	Different
Class 2	Same	Same	Same	Same	Same	Yes	Yes	Different

### Instruments

#### Test

In the empirical experiment teaching, Class 1 as the control group followed the traditional teaching model, while Class 2 as the experiment group adopted the IVRTM for a term. Their CEE acted as a pre-test and the final exam was a post-test. All 109 participants took the final exam after a one-term teaching experiment, and the scores would be collected and analyzed to test the significance of the experiment results. SPSS was used for data statistics and analysis in the research. Sig. value < 0.05 was considered statistically significant.

#### Questionnaire

In order to acquire the students’ attitudes toward the IVRTM and the application of VR technology in teaching, the researcher designed and adopted a questionnaire to investigate their acceptability of the IVRTM and VR technology used in teaching.

#### Interviews

The researcher made an interview with five students selected randomly from the experiment group who thought the IVRTM couldn’t decrease their learning anxiety. Open questions were also available for the students’ views on the IVRTM.

Q1: What do you think of the IVRTM?
Q2: Why did you think the new teaching couldn’t decrease your learning anxiety?

#### Big data analysis of learning platforms

Big data on the learning platforms were used for research on the students’ academic performance in the learning process. There were numerous Big Data available on the learning platforms related to the students’ learning situations, such as online study duration, completion rates, and numbers of posts, sharing, and discussions, which could be used as references for educational management, assessment, and research (Zhang and Li, 2013). The procedural evaluation of pre-class, in-class, and after-class learning would be of importance. An analysis of qualitative statistics was also used for students’ attendance and completion rates of learning tasks and the feasibility and acceptability of the IVRTM.

### Implementation of the immersive virtual reality teaching model

**FIGURE 3:**
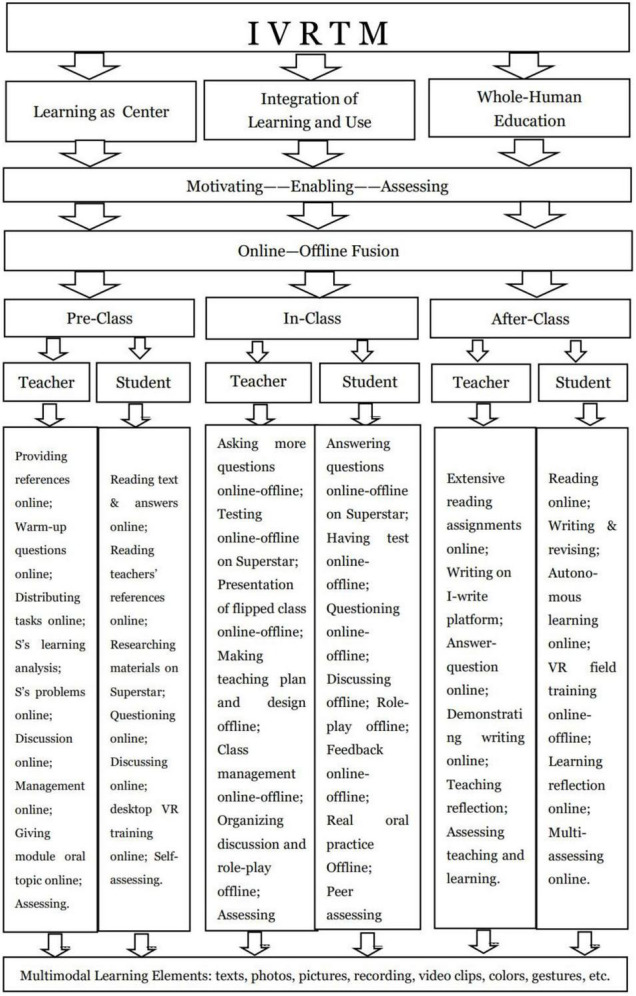
The flowchart of the POA theory.

The teaching procedures of the IVRTM has three sections, including pre-class output-oriented prevision, in-class tasks-driven and problem-driven presentation and after-class revision and writing demonstrations. The Flowchart of the POA Theory (Figure 3) represents the teaching procedures and activities of the IVRTM.

Pre-class learning as an actual preparation for further study was performed online on the learning platform called Superstar Learning Platform, referred to as Superstar. The learning platform has some functions and a lot of learning resources. Generally, it has five functional sessions:

(1) Pan construction course for building courses and uploading learning resources;
(2) Online output-led and task-driven teaching activities;
(3) Projection screen teaching for screen teaching and activate classroom activities;
(4) Diverse assessment, especially an emphasis on procedural evaluation;
(5) Learning management.

It provided the students with book-related background information, questions, exercises, tests, and extensive activities helpful for their pre-class preparations, in-class interactive activities, and after-class revision. The activities consisted of text-related warm-up questions, exercises, and audio-visual clips, which fully mobilized students’ auditory, visual and tactile perceptions in the forms of listening, watching, speaking, reading, writing, translating, and imitating. The students had an all-round educational experience on the multimodal learning platform. Since university environments had diverse student populations with a wide variety of learning styles, the multimodal activities helped each student achieve their personalized learning experience on their own, which followed the POA philosophy of Learning as Center and students as the main learning body. Selective learning concerned the students’ personalized learning characteristics according to students’ different levels, orientations, and styles to achieve learning objectives and improvement of each student. During the practice, the students were motivated by the output objective, integrating learning into use to achieve the learning goal. In the pre-class online learning process, the teacher was in charge of references sharing, tasks attribution, assignments, analysis of students’ proficiency, problem collection, online teaching and learning management, oral topic release and data collection for assessment. He led and instructed the students’ automatous learning online regularly if necessary. The students would preview the text and related words and expressions. And they could scan the QR code to get the audio recording of them. Besides, there were many audio-visual materials for exercises and references. More tasks include answering the warm-up questions, putting forward questions and challenges, and discussing problems with the teacher and other students on the forum of Superstar. VR oral desktop training could be done before class. Pre-class learning mainly depended on online learning. I-learning data was for assessment, which was helpful for the mastery of the students’ learning process and strengthening of procedural assessment (Milicevic et al., 2017). Cooperative learning occurred when the students have some problems, they would post and discuss with their peers and the teacher on the discussion forum, which gave full play to the wisdom of the collective (Cumming, 2001; Rubin, 2005; Zhan, 2019).

In-class teaching was organized in the form of flipped classrooms with face-to-face and online–offline fusion teaching depending on the Superstar Platform in the class. The teacher used a projection screen for teaching and activated learning activities on the Yu Classroom of Superstar. He checked out the achievement of students’ prevision by asking questions and a short quiz, made a presentation for major and difficult points, answered the students’ questions, resolved their problems, organized discussion and role-play, and evaluated learning and teaching. The students answered the teachers’ questions, completed quiz, discussed with peers, made role-plays, watched, listened to and feedbacked the teacher’s presentation, put forward questions, and evaluated one another. The major points were often presented and interpreted through questions and PPTs in the flipped classroom (Wang et al., 2021). However, the IVRTM was totally different from the traditional teaching of PPTs presentation and interpretation one point after another. In the IVRTM, the teacher had no necessity to cover each point, some of the major points or difficult points in the flipped class were emphasized because the teacher had known what the students mastered according to their performance in pre-class tasks, questions, and discussion.

In class, the teacher adopted the following online-offline fusion teaching procedures. For instance, one or two students could be asked to answer the teacher’s questions on the blob, while other students could post their answers on the platform forum. Sometimes they could thumb-up some excellent answers on the screen, and the teacher gave instant and interactive online-offline feedback. The students’ posts would be recorded as their assessing references for classroom participation. What’s more, group discussion was helpful for stimulating the students’ imagination, cultivating their cooperative spirit, and improving their thinking ability. They usually brought out possibilities that individual members of the group had not thought of Cumming (2009). Students were divided into several groups to discuss a topic related to the theme of the text, and the teacher as the teaching subject organized the discussion and gave them some necessary instructions and suggestions. After that, they were asked to practice the oral topic that had been given in pre-class by role-playing. And they had practiced similar situations on the desktop VR training. Ideas and values were conveyed through topic discussion, mutual assistance, and cooperation in the group activities as well as role-play (Wang et al., 2021). The philosophy of Whole-Human Education aimed for the cultivation of students’ comprehensive quality, such as teamwork cooperation, innovation, and autonomous learning ability. During the learning process, the students integrated output–input, making the philosophy of Learning and Use possible. Moreover, multimodal learning changed teaching and learning styles using multiple information means and channels, including audio, speech, music, pictures, text, illustrations, imitation, writing, gestures, facial expressions, and colors. The students’ perceptions were fully activated by speaking, listening, watching, discussing, questioning, and assessing in the learning process.

After-class revision aimed at the students’ to consolidate what they had learned in the module. The teacher did a lot, including assignments (compulsory writing and selective tasks), online answer-question instruction, extensive materials sharing online, the reflection of teaching, and assessment. The writing was completed on the I-write Platform, a professional academic writing platform. The students revised their essays several times and got revising comments and different marks through computer evaluation. The repeated revision could improve the students’ writing level greatly according to the scores. The highest marks would be acted as the final assessment scores of the writing. Besides, both the teacher and peers’ evaluation on the platform could be done, too. Compared with the traditional teaching writing—handing in for the teacher’s comments on the composition, the academic writing was more effective and efficient. Moreover, the traditional writing was graded and written only once, and usually assessed by the teacher. After that, the teacher often selected one or two excellent papers to illustrate how to organize writing. Multi-evaluation was adopted to promote the fairness of evaluation and encouraged students’ participation in assessment, such as machine evaluation, teacher’s evaluation, peers’ mutual evaluation, and student’s self-evaluation (Cumming, 2009). What’s more, the teachers gave some instructions on writing online by demonstrating a few excellent compositions online (Chang, 2019). VR field training in the Smart Room in the students’ spare time, which was open regularly. They could experience virtual-reality interaction in the immersive space.

More importantly, there are Big Data available on the learning platforms related to the students’ online study duration, completion rates, frequency of posts, sharing, and discussions, which can be used as references for educational management, assessment, and research. For instance, teachers could monitor and instruct each student online and give him/her some suggestions on learning methods and learning progress according to the Big Data. In addition, teachers could check out students’ class engagement according to the Big Data. Moreover, teachers could set up the proportion of scores for each task. Generally, Big Data of platforms were helpful for procedural evaluation, and what students have done would be quantified and assessed in the whole learning process (Jayashankar, 2018). To date, data analytics has been used in education, and automated and scalable analytics tools with synchronous data would promote Big Data in teaching and learning management and evaluation greatly (Cumming, 2009).

## Results and analysis

### Results and analysis of pre-test

In the pre-test, according to the result of the Shapiro–Wilk test in Table 2, the values of sig. are 0.159 and 0.942, and they were both larger than 0.05, which were not considered statistically significant. It indicated that two sets of data were normally distributed.

**TABLE 2:** Normality assumption test for pre-test.

		Shapiro–Wilk test		
	Class	*F*	df	Sig.
Pre-test	1	0.990	53	0.942
	2	0.969	56	0.159

Table 3 showed that the mean scores of the two classes were 67.25 and 67.18, which were similar from the perspective of descriptive statistics. According to the results of Levene’s test of variance, when *p* = 0.003 < 0.05, the equal variance was not assumed. When Sig. (two-tailed) value = 0.969 > 0.05, there was no statistical difference in the performance of the two classes, that is, they had similar English mastery levels before the experiment.

**TABLE 3:** Independent sample test of pre-test.

MS		SD		*F*	*P*	*t*	*f*	Sig. (two-tailed)	95% CI	
Class 1	Class 2	Class 1	Class 2						Lower	Upper
67.251	67.180	7.463	11.340	9.483	0.003	−0.039	108	0.969	3.7022	3.561

MS, mean scores; SD, standard deviation; CI, confidence interval.

### Results and analysis of post-test

Descriptive and qualitative statistics of the final exam results were used to identify the significance of the IVRTM (Table 4). The statistics of post-test were shown as follows.

**TABLE 4:** Normality assumption test for post-test.

		Shapiro–Wilk test		
	Class	*F*	df	Sig.
Post-test	1	0.980	53	0.493
	2	0.979	56	0.433

In the post-test, the values of sig. were 0.493 and 0.433, both larger than 0.05 according to the results of the Shapiro–Wilk test in Table 4, which was not considered statistically significant. The test proved the fact that the two sets of data were normally distributed.

Table 5 showed that the mean scores of the two classes were 67.81 and 73.146. The scores of Class 2 were higher than those of Class 1 from the perspective of descriptive statistics. According to the results in the table, *P* = 0.001 < 0.05, which proved the fact that there was statistical difference in the performance of the two classes, that is, there was obvious significant difference between the scores of the two. In the experiment, the two groups had the same teacher, periods, books, final exam, and teaching plan except for the teaching methods. Therefore, it could be concluded that the difference in the exam scores between the two classes resulted from the different teaching models. That is to say, the IVRTM with learning platforms and VR assistance in the SLA teaching was obviously better than the traditional teaching method.

**TABLE 5:** Independent sample test of post-test.

MS		SD		*F*	*P*	*t*	*f*	Sig. (two-tailed)	95% CI	
Class 1	Class 2	Class 1	Class 2						Lower	Upper
67.819	73.146	6.641	10.832	11.519	0.001	3.114	108	0.002	1.930	8.762

MS, mean scores; SD, standard deviation; CI, confidence interval.

### Results and analysis of recording text in oral English practice

In the last oral English practice of the term, the students of the two classes were asked to do role-plays and complete an oral topic of the same situation within 3 min. All of the dialogues were recorded, and the data of recording text were taken statistically and analyzed (Table 6). Mean scores were from the average score of teacher’s evaluation, peer’s evaluation, and machine evaluation.

**TABLE 6:** Analysis of oral English recording text.

Class 1	Class 2	Items
6.24	7.09	Mean scores
17.1	25	Interaction frequency
97.36	109.18	Speech speed
2.15	2.81	Average interaction duration
12.21	12.27	Average sentence length
13.4%	18.27%	Words used in Band 4 College English Test Vocabulary

According to the statistics, the results of the comparative data indicated Class 2 did better than Class 1 in all of the aspects, including mean score, interaction frequency, speech speed, average interaction duration, average sentence length, and words ratio used in Band 4 College English Test vocabulary, in spite of the gap of average sentence lengths not particularly significant. From the data, the blended teaching with VR assistance promoted the students’ communicative ability.

### Results and analysis of questionnaire

The questionnaire on the students’ attitudes toward the IVRTM was performed among the experiment group, and the statistics are shown in Table 7.

**TABLE 7:** Data statistics of questionnaire survey.

Items	Questions	Acceptability index
1	Be able to improve language competence	83.64%
2	Be able to improve learning enthusiasm	72.73%
3	Be able to improve learning efficiency	94.50%
4	Be able to improve learning engagement	100%
5	Be able to decrease learning anxiety	56.36%
6	Approval of using VR-assisted Smart Class	100%
7	Better than traditional teaching model	80%

According to Table 7, the proportions of acceptability were 83.64, 72.73, 94.5, and 100% separately for the improvement of language competence, learning enthusiasm, learning efficiency, and learning engagement. As for Item 5 “be able to decrease learning anxiety,” 56.36% of students took a positive attitude toward it. According to the statistics of Item 7, 80% of students accepted the IVRTM adopted in the SLA teaching and thought that it was better than the traditional teaching model. They preferred VR-assisted teaching in the online-offline fusion ecological environment.

As for the open question “Is the IVRTM conducive to improving your autonomous learning ability,” 44 out of 56 students answered it positively; 80% of students thought that they searched materials on the internet, grouped them on their own, and shared them with their classmates, which were beneficial to cultivate their autonomous learning ability. What’s more, they discussed some questions with others online. They said that the learning model was completely different from the traditional one. They just had completed some written homework assigned by their teachers before. On the contrary, the IVRTM forced them to search for a lot of learning materials, sort them out and think independently. About 80% of the students accepted the IVRTM completely and thought that the flipped classroom in the IVRTM fully mobilized their learning enthusiasm and autonomy, and multimodal tasks improved their English comprehensive competence. And 20% of them thought of it oppositely. They claimed that online-searching information was time-consuming and laborious. They admitted that they sometimes drifted down to unrelated links uncontrollably and suggested that more supervision was needed in their learning process.

For another question “What do you think of VR technology used in the IVRTM,” 100% of students thought that VR-assisted learning offered them a completely different learning experience, full of novelty and interest in learning. In addition, they could select some tasks for personalized learning. They also pointed out that the IVRTM had some positive effects on them. For example, compared with the previous relaxing learning style, they are accustomed to prevision in pre-class. If there were some problems, they would think independently and discuss with peers to discover the solutions to the problems rather than entirely relying on their teachers. They changed their learning concept from “learning how to learn” to “learning autonomously” (Fiore and Rosenquest, 2010). They admitted that the IVRTM needed more self-discipline and effort definitely.

### Results and analysis of big data on the platform

There were some Big Data OF the learning platforms collected and analyzed as follows (Table 8).

**TABLE 8:** Statistics of big data.

	Attendance rate	Study duration (weekly)	Discussion frequency (per period)	Question frequency (per period)	Share frequency (per period)
Class 1	82.8%	0.8–1 h	0.26 times	0.13 items	0.12 times
Class 2	98.5%	10.12–12.78 h	5.41 times	3.74 items	4.23 times

According to Table 8, the student’s attendance rate (98.5%) in Class 2 was obviously higher than that (82.8%) in Class 1, and it was the same in other aspects, such as study duration, discussion frequency, question frequency, and share frequency. The results of descriptive statistics indicated that the students in Class 2 were more engaged in the learning than those in Class 1 obviously. Therefore, a reasonable interpretation of the difference would contribute to the different teaching method—IVRTM, which activated the students’ multimodal perceptions and learning enthusiasm for higher learning effectiveness. The students were motivated to work hard and efficiently under the guidance of the IVRTM in the VR-assisted spatial fusion learning environment. Most of the students in the experiment group accepted the IVRTM and made some progress in their academic performance. More importantly, the IVRTM had an effect on their learning styles, especially effective time management, learning cooperation, and autonomous learning.

### Results and analysis of interviews

The researcher made an interview with five students about the IVRTM and the reduction of learning anxiety. The following is the recording of their interviews.

*Participant 1*: *Of course, the VR-assisted teaching itself can lower our anxiety in oral practice. However, the pre-class, in-class and after-class preparations need more efforts. I spend much more time on autonomous learning, which puts a great pressure on me.*
*Participant 2*: *The VR-assisted learning offers me an interactive learning environment, full of real-time feedbacks and dynamic personalized learning. But I feel anxious. And sometimes the situation is counter-productive. It is difficult for me to keep up with the teaching schedule and complete all of the tasks because of my low English proficiency.*
*Participant 3*: *The IVRTM requires us to study independently and the fusion learning environment promotes me to work actively. In practice, I am forced to improve my time management skills and adapt myself to the new learning role from a passive knowledge recipient to an autonomous learner.*
*Participant 4*: *I think I should overcome some bad learning habits, like delay of homework, to keep up with learning schedule. I’m lack of self-discipline, texting to my friends and playing games online instead of learning English occasionally.*
*Participant 5*: *The IVRTM gives me a new experiential learning by using a new technology. VR assistance in teaching as an “easy to use,” “helpful,” and “enjoyable” tool* (Shen, 2021) *can reduce my psychological anxiety when practicing oral English. However, I still had a little interactive anxiety in real face-to-face situations. I guess it would be better after a longer time practice.*

To sum up, the students consistently admitted that VR-assisted could lower their anxiety definitely in oral English practice. They had a visual-real experience in scenario dialogue in an IVR setting, in which they experienced a “real” situation without any anticipatory anxiety as it was in the real one. The visual practice made them relaxed when making a human-computer conversation without face-to-face awkwardness in reality. The virtual characters taught them to use correct and accurate speech and proper communicative skills and awareness of cultural differences. In real situations, they would make use of the correct speech that they had acquired from the virtual situations.

As for the issue of decreasing anxiety with a low approval rate (56.36%), the students admitted that the pressure mainly came from the whole learning process instead of VR-assisted learning itself. Thanks to VR assistance, they had less communicative anxiety encouragingly. Learning stress and anxiety were due to the compacted online-offline learning arrangement under the guidance of the IVRTM. Therefore, good time management and autonomous learning habits were helpful for their catching up with the learning schedule.

## Conclusion

This study investigated the effectiveness of the teaching design scheme called the IVRTM on a small learner population at the researcher’s university. The researcher made a teaching design of a spatial fusion environment, extending the physical classroom to a 3-layer ecological class involved in classroom teaching, online teaching, and VR-assisted teaching. It aims for the effectiveness of the IVRTM in a spatial fusion ecological class, uncovering the VR-assisted positive effect on oral English practice, and acquiring the students’ attitudes toward the IVRTM. 109 participants in the empirical study completed the learning tasks over a term. The study approaches were adopted including literature review, empirical method, questionnaire, interviews and data statistics, and analysis. There were some findings as follows.

### Findings

(1) The IVRTM was better than the traditional teaching method, which activated the students’ multimodal perceptions to acquire higher learning efficiency. The research would have a positive significance for the promotion of new technology applications in SLA teaching. It provided novel and preliminary references for teachers pursuing an effective teaching design of ecological class based on IVR spatial fusion, for there were few research on the combination of online-offline fusion with VR assistance. According to the questionnaire, the IVRTM was accepted by the majority of the students about 80% (44 out of 56) in Class 2, which indicated the fact that the students in the experiment group generally recognized the advantages of the IVRTM. What’s more, the students’ high learning engagement indicated the acceptability of the IVRTM, such as attendance rate, learning duration, and frequencies of posts, discussions, and questions. And the students made significant progress in the final exam and gained development in several aspects, like autonomous learning, cooperative learning, and independent thinking according to the interviews. It was true that the IVRTM enhanced high feasibility and acceptability in the experiment group, which implied its good prospect of further promotion in the future.
(2) Through the research on the recording text of the students’ oral practice, the participants in the experiment class performed better in all aspects, such as mean scores, interaction frequency, speech speed, average interaction duration, average sentence length, and words ratio of Band 4 College English Test vocabulary. VR assistance had a positive and significant effect on the students’ spoken competence. According to Item 6 of the questionnaire, about 80% of students accepted and welcomed the application of VR technology in teaching.
(3) Moreover, the students in Class 2 gained more learning styles, time management methods, autonomous learning habits, and novel thinking ways according to the interviews. The cultivation of comprehensive literacy in Whole-Human education was regarded as one of the most important advantages of the IVRTM.
(4) VR-assisted learning could reduce the students’ anxiety, especially in oral practice. Although the procedural learning of the IVRTM was tense and compacted, VR-assisted learning made them relaxed. According to the interview, the VR-assisted training avoided face-to-face interactions due to awkward incorrect language usage and use among peer students. VR digital technology could activate the students’ multisensory modes for language acquisition (Hu et al., 2021), which fully mobilized their vision, hearing, taste, smell, and other senses to complete language acquisition in the novel, creative and immersive space, experiencing the interactive situations with “real presence” (Rubin, 2005). VR-assisted learning promoted the students’ interest and enthusiasm, beneficial to learning efficiency in the SLA.

### Limitations

There were several limitations of the study.

(1) The safety of online information should be taken into consideration. It is important for the students to establish an awareness of safety on the internet, especially in the application of new technology in the future.
(2) Learning duration online is another important issue. Long-time online learning may be harmful to the students’ health as a result of the diminution of vision, hypoplasia, and headache. So, limiting online learning time could avoid some negative effects on young people.
(3) Further enlarging the study samples in scope and quantity would make the experiment results more valid and reliable, for only 56 students were recruited to participate in the experiment in the study. More samples should be selected from different grades and colleges to test the validity of the study. Therefore, a wider range of students would be recruited for the future study.
(4) Financial support was necessary for the IVRTM promotion, for it needs a great deal of investment in devices, like Smart Class with VR equipment, projects, and related digital technology.
(5) Last but not least, it was a lack of further duplicated IVRTM empirical teaching in the control group due to the time limit. More duplicated teaching experiments for different samples would be followed and more findings gained in the further research, which would provide more references for the validity and reliability of the IVRTM.

### Discussion

This study created the IVRTM and verified its significant teaching effectiveness among a small learner population of the two different classes. The students could get higher learning effectiveness under the guidance of the IVRTM based on POA theory. It uncovered the positive effect of VR and digital ecological technology in the SLA teaching in an ecological environment based on spatial fusion. VR- assisted teaching in oral English practice promoted the students’ communicative competence. Most of the students (about 80%) accepted the IVRTM and new technologies used in teaching and learning according to the questionnaire. Multimodal teaching was helpful to stimulate learners’ visual, auditory and tactile perceptions by listening, speaking, reading, writing, and imitating for improving learning efficiency. The research results supported and authenticated all the previous hypotheses of the study, answered the research questions raised in the preface and achieved the stated research goal.

Combined with the relevant literature references, the research results in this study were consistent with those in their studies. For instance, the construction of ecological classes and a smart learning environment could make learning and teach more effective (Zhen, 2017; Xie, 2019). Big Data science and learning analytics in education made educators’ master learners’ learning status more convenient, especially helpful for the procedural learning management (Milicevic et al., 2017).

The IVRTM has broad research prospects in the learning process, learning management, and learning psychology. Big Data on the learning platform could be used in a variety of fields, gradually covering data analysis, education policy guidance, learning model development, personalized demand analysis, and application iteration (Jayashankar, 2018). VR assistance in the interdisciplinary cooperation mechanism also greatly promotes the narrative communication of the metaverse. It can also be viewed that each educational innovation needs thinking about how to realize the interconnection of various mass media in the embodied and digital times. A new chapter of multi-spatial education with new technologies is open in the new times. Take multimodal teaching, for example, research on photos, pictures, texts, etc., will be beneficial to learners’ emotional changes in the learning process. Of course, there are still limitations of the IVRTM mentioned above, such as distraction from computer games, internet safety, and further research in depth and width. Not all schools have enough investment in smart teaching, so it is important to raise more money for education and achieve educational equality. In addition, the mastery of teachers’ educational technologies is also very important, and educational technology training plays a key role in teachers’ professional growth.

The study was based on one-term experimental teaching and uncovered the positive impact of the ecological environment. The research proved the fact that the IVRTM was better than the traditional class for higher learning effectiveness. Digital and VR technologies were helpful to stimulate learners’ visual, auditory and tactile perceptions for promotion of learning efficiency (Yang and Gou, 2020) as well as students’ comprehensive literacy (Zhang, 2011). Data analysis enabled teachers to comprehensively evaluate students’ academic performance, giving them feedback and personalized instruction (Zhang and Li, 2013). The study made significant contributions to multi-spatial fusion teaching combining online–offline English teaching with VR assistance through its unique teaching model, which would have a novel and positive significance for the promotion of new technologies in education. The findings would be strong proof that the IVRTM was an effective teaching model of ecological class based on spatial fusion.

In the future, the “AI + Education” model will be integrated into multiple disciplines and a combination of multidisciplinary research, education management, and educational evaluation with educational technology. Furthermore, multi-linguistic research will become the trend of the SLA with the development of diversification in the world (Wen and Wang, 2004; Zhang, 2011). Faced with the new situations, teachers’ training is demanding and urgent, not only in educational technology but also in educational concepts (Marsh and Boag, 2013). The online-offline hybrid and technology-enhanced teaching and learning model breaks through the physical boundary and extends a three-layer fusion space. An ecological learning environment based on multi-space fusion is demanding in future education development (Zhang, 2011). Informatization, digitalization, and virtualization will constantly deepen technological application to promote the integration of smart teaching, smart management, smart evaluation, smart research, and Smart equipment in educational innovation (Xie, 2019).

## Data availability statement

The original contributions presented in this study are included in the article/supplementary material, further inquiries can be directed to the corresponding author.

## Ethics statement

Ethical review and approval was not required for the study on human participants in accordance with the local legislation and institutional requirements. Written informed consent from the patients/participants or patients/participants legal guardian/next of kin was not required to participate in this study in accordance with the national legislation and the institutional requirements.

## Author contributions

The author confirms being the sole contributor of this work and has approved it for publication.

## Conflict of interest

The author declares that the research was conducted in the absence of any commercial or financial relationships that could be construed as a potential conflict of interest.

## Publisher’s note

All claims expressed in this article are solely those of the authors and do not necessarily represent those of their affiliated organizations, or those of the publisher, the editors and the reviewers. Any product that may be evaluated in this article, or claim that may be made by its manufacturer, is not guaranteed or endorsed by the publisher.

## References

[B1] Anua M. Ismail I. Shapri N. S. Mat Amin M. Arsad M. (2022). A systematic review of purpose and latency effect in the virtual reality environment. *Int. Technol. Int. Entert.* 429 403–413. 10.1007/978-3-030-99188-325

[B2] Chacon A. Chacon M. Mas M. Avargues Navarro M. Luisa A. (2021). Cross- cultural adaptation and validation of the highly sensitive person scale to the adult spanish population (HSPS-S). *Psychol. Res. Behav. Manage.* 14 1041–1052. 10.2147/PRBM.S321277 34285606 PMC8286783

[B3] Chang R. (2019). Promotion of practical exploration in college English writing teaching based on the “production-oriented approach”. *Educ. Moderniz.* 13 133–135. 10.16541/j.cnki.2095-8420.2019.13.043

[B4] Cumming A. (2001). Learning to write in a second language: two decades of research. *Int. J. English Stud.* 1 1–23. 10.6018/ijes.1.2.48331

[B5] Cumming A. (2009). What needs to be developed to facilitate classroom-based assessment. *TESOL Quart.* 43 515–519.

[B6] Duan D. P. (2019). Research summary of application of VR in education from various theoretical perspectives. *China Educ. Technol. Equipment* 456 54–60. 10.3969/j.issn.1671-489X.2019.06.054

[B7] Fiore L. Rosenquest B. (2010). Shifting the culture of higher education: influences on students, teachers, and pedagogy. *Theory Into Pract.* 49 1–14. 10.1080/00405840903435535

[B8] Hu Y. L. Chang X. Y. Wu B. (2021). The influence of immersive virtual-reality on skill transfer: the moderating effect of learning style. *J. Dis. Educ.* 39 63–71. 10.15881/j.cnki.cn33-1304/g4.2021.02.007

[B9] Jayashankar M. S. (2018). Big data analytics for rapid, impactful, sustained, and efficient (RISE) humanitarian operations. *Prod. Operat. Manage.* 27 1696–1700. 10.1111/poms.12840

[B10] Li D. Fan Y. X. (2018). Designing a lecture for the language enabling phase of POA teaching process. *Mod. Vocat. Educ.* 25 76–78. 10.3969/j.issn.2096-0603.2018.25.036

[B11] Marsh T. Boag S. (2013). Evolutionary and differential psychology: conceptual conflicts and the path to integration. *Front. Psychol.* 4:655. 10.3389/fpsyg.2013.00655 24065949 PMC3779804

[B12] Milicevic K. Ivanovic A. Budimac M. (2017). Data science in education: big data and learning analytics. *Comput. Appl. Eng. Educ.* 25 1068–1078. 10.1002/cae.21844

[B13] Rubin J. (2005). Achieving success in second language acquisition. Betty Lou Leaver, Madeline Ehrman, and Boris Shekhtman. *Stud. Second Lang. Acquisit.* 29:637. 10.1017/S0272263107070581

[B14] Shen J. P. (2021). A review of the effectiveness of foreign language enjoyment and foreign language classroom anxiety on learners’ engagement and attainment. *Front. Psychol.* 12:1–4. 10.3389/fpsyg.2021.749284 34552544 PMC8450351

[B15] Wang W. F. Mao M. J. (2021). Multimodal learning analytics: a new approach to understand and evaluate authentic learning. *Res. Electr. Educ.* 334 33–39. 10.13811/j.cnki.eer.2021.02.004

[B16] Wang Y. L. Derakhshan A. Zhang J. L. (2021). Researching and practicing positive psychology in second/foreign language learning and teaching: the past, current status and future directions. *Front. Psychol.* 12:1–10. 10.3389/fpsyg.2021.731721 34489835 PMC8417049

[B17] Wen Q. F. (2015). Developing a theoretical system of “production-oriented approach” in language teaching. *Lang. Teach. Res.* 47 547–558.

[B18] Wen Q. F. (2016). Teaching culture(s) in English as a lingua franca in Asia: dilemma and solution. *J. English Ling. Franca* 5 155–177.

[B19] Wen Q. F. (2018). Production-oriented approach and overseas Chinese teaching. *World Chin. Teach.* 32 387–400. 10.13724/j.cnki.ctiw.2018.03.008

[B20] Wen Q. F. (2021). On the role of language in international academic discourse power: the status of Chinese. *Chin. J. Lang. Policy Plan.* 6 76–85. 10.19689/j.cnki.cn10-1361/h.20210306

[B21] Wen Q. F. Wang L. F. (2004). SLA research methods over 35 years: looking back and ahead. *J. Foreign Lang.* 152 18–25.

[B22] Xie Y. Z. (2019). Discussion on effective coupling between smart learning environment and task-driven teaching. *China Educ. Technol. Equipment* 24 44–46.

[B23] Yang C. Gou S. H. (2020). An overview of the POA (2015-2019): retrospect, current status and prospect. *J. Ningbo Univ. Technol.* 32 82–89. 10.3969/j.issn.1008-7109.04.015

[B24] Zhan X. (2019). Adaptation of German textbooks based on the POA: promote the procedural design of activities. *Foreign Lang. Their Teach.* 304:145. 10.13458/j.cnki.flatt.004556

[B25] Zhang B. C. (2011). Studies of humanistic education in China’s foreign language education: current situation, limitation and future direction. *Educ. Teach. Res.* 25 34–46. 10.13627/j.cnki.cdjy.2011.07.003

[B26] Zhang Y. Li Y. (2013). Learning analytics and education assessment based on big data generated by MOOCs. *Tsing. J. Educ.* 34 22–26. 10.3969/j.issn.1001-4519.2013.04.004

[B27] Zhen Z. (2017). The model construction of English ecological class in the high school in China. *English Lang. Teach.* 10 227–231. 10.5539/elt.v10n9p227

[B28] Zhu Y. Bai X. (2019). Application of production-oriented approach in Chinese teaching: the achievement of output goal. *World Chin. Teach.* 33 95–103. 10.13724/j.cnki.ctiw.2019.01.009

